# Adaptive lymphocyte profiles correlate to brain Aβ burden in patients with mild cognitive impairment

**DOI:** 10.1186/s12974-017-0910-x

**Published:** 2017-07-27

**Authors:** Ann M. Stowe, Sara J. Ireland, Sterling B. Ortega, Ding Chen, Ryan M. Huebinger, Takashi Tarumi, Thomas S. Harris, C. Munro Cullum, Roger Rosenberg, Nancy L. Monson, Rong Zhang

**Affiliations:** 1 Department of Neurology and Neurotherapeutics, UT Southwestern Medical Center, NL9.110E, 6000 Harry Hines Blvd, Dallas, 75390 TX USA; 2 Department of Surgery, UT Southwestern Medical Center, 6000 Harry Hines, Dallas, 75390 TX USA; 3 Department of Radiology, UT Southwestern Medical Center, 6000 Harry Hines, Dallas, 75390 TX USA; 4 Department of Psychiatry, UT Southwestern Medical Center, 6000 Harry Hines, Dallas, 75390 TX USA; 5 Department of Immunology, UT Southwestern Medical Center, 6000 Harry Hines, Dallas, 75390 TX USA; 6 Texas Health Presbyterian Hospital, Institute for Exercise and Environmental Medicine, 7232 Greenville Ave, Dallas, 75231 TX USA

**Keywords:** Amnestic mild cognitive impairment, Amyloid burden, ^18^F-florbetapir, Memory B cells, IgG, T lymphocytes, CD4 T cells, Cerebrospinal fluid

## Abstract

**Background:**

We previously found that subjects with amnestic mild cognitive impairment exhibit a pro-inflammatory immune profile in the cerebrospinal fluid similar to multiple sclerosis, a central nervous system autoimmune disease. We therefore hypothesized that early neuroinflammation would reflect increases in brain amyloid burden during amnestic mild cognitive impairment.

**Methods:**

Cerebrospinal fluid and blood samples were collected from 24 participants with amnestic mild cognitive impairment (12 men, 12 women; 66 ± 6 years; 0.5 Clinical Dementia Rating) enrolled in the AETMCI study. Analyses of cerebrospinal fluid and blood included immune profiling by multi-parameter flow cytometry, genotyping for apolipoprotein (APO)ε, and quantification of cytokine and immunoglobin levels. Amyloid (A)β deposition was determined by ^18^F-florbetapir positron emission tomography. Spearman rank order correlations were performed to assess simple linear correlation for parameters including amyloid imaging, central and peripheral immune cell populations, and protein cytokine levels.

**Results:**

Soluble Aβ_42_ in the cerebrospinal fluid declined as Aβ deposition increased overall and in the precuneous and posterior cingulate cortices. Lymphocyte profiling revealed a significant decline in T cell populations in the cerebrospinal fluid, specifically CD4+ T cells, as Aβ deposition in the posterior cingulate cortex increased. In contrast, increased Aβ burden correlated positively with increased memory B cells in the cerebrospinal fluid, which was exacerbated in APOε4 carriers. For peripheral circulating lymphocytes, only B cell populations decreased with Aβ deposition in the precuneous cortex, as peripheral T cell populations did not correlate with changes in brain amyloid burden.

**Conclusions:**

Elevations in brain Aβ burden associate with a shift from T cells to memory B cells in the cerebrospinal fluid of subjects with amnestic mild cognitive impairment in this exploratory cohort. These data suggest the presence of cellular adaptive immune responses during Aβ accumulation, but further study needs to determine whether lymphocyte populations contribute to, or result from, Aβ dysregulation during memory decline on a larger cohort collected at multiple centers.

**Trial registration:**

AETMCI NCT01146717

**Electronic supplementary material:**

The online version of this article (doi:10.1186/s12974-017-0910-x) contains supplementary material, which is available to authorized users.

## Background

Alzheimer’s disease (AD) is a complex illness [1, 2], influenced by both environmental and genetic risk factors [3, 4]. Amyloid (Aβ) burden increases with normal aging [5], but extensive Aβ plaque depositions are a hallmark of AD, which may trigger a pathological cascade resulting in neuronal injury and cognitive impairment (i.e., Aβ hypothesis) [6, 7]. Under physiologic conditions, neuronally derived Aβ_42_ is balanced between production and clearance [8], as soluble Aβ_42_ in the extracellular space is cleared by proteolysis, paravascular pathways, and blood-brain barrier transporters [9–11]. In AD, reduced clearance from the brain may result in a ~30% reduction of Aβ_42_ detected in the CSF of subjects with late-onset AD compared with healthy subjects [8].

Inflammation (e.g., activated microglia, reactive astrocytes, pro-inflammatory cytokines) is also commonly found in AD brains [12, 13] and attributed to a potential immunogenicity of misfolded amyloid Aβ. This inflammatory response to pathological Aβ accumulation likely occurs decades prior to AD-associated cognitive decline [14], but whether this response contributes to or is associated with amyloid accumulation is unknown. We recently showed that CD45^+^ leukocytes and pro-inflammatory cytokines in the cerebrospinal fluid (CSF) of subjects with amnestic mild cognitive impairment (aMCI), at a high risk for developing AD [15, 16], are present at levels equivalent to patients at the earliest stage of multiple sclerosis (MS), a central nervous system (CNS) autoimmune disease, confirming the presence of CNS inflammation [17]. These data suggest that aMCI patients display features of CNS inflammation, although the impact this may have on brain amyloid deposition is unknown.

The purpose of this study was to investigate a potential relationship between adaptive immune cell populations and brain amyloid deposition. We assessed the profile of adaptive immune cells in the CSF, which is in close proximity to the brain, in contrast to previous studies that focused on the peripheral blood [18–22]. Thus, we quantified CSF- and blood-derived immune profiles by flow cytometry and ELISA, brain amyloid (Aβ) deposition by ^18^F-florbetapir positron emission tomography (PET) [23], and soluble Aβ_42_ in the CSF by standard ELISA. We found that the extent of Aβ deposition correlated with a shift towards reduced frequencies of T cells and increased frequencies of memory B cells in the CSF. Additionally, CSF levels of the cytokine IL-6 correlated inversely with Aβ deposition. These data suggest that it is critical to identify novel risk factors that presage maladaptive immune mechanisms during AD [17], as they hold the potential to become important immunotherapeutic targets for early intervention [24]. These findings should be confirmed in a larger cohort collected at multiple centers.

## Methods

All subjects signed written informed consent approved by the Institutional Review Boards of the UT Southwestern (UTSW) Medical Center and Texas Health Presbyterian Hospital of Dallas, Texas. The subjects (65.7 ± 6.0 years) were enrolled in a larger exercise study but gave consent for lumbar puncture, blood draw, and/or PET imaging at baseline prior to randomization in the exercise study. A 1:1 male-to-female ratio (12 males and 12 females) was maintained to reduce the confounder of sex bias. Six subjects did not have banked cells for apolipoprotein (APO)ε genotyping. All subjects underwent clinical interviews and a standard battery of neuropsychological tests to establish clinical diagnosis via multidisciplinary consensus using standard criteria by a licensed psychometrician at UT Southwestern. Detailed inclusion and exclusion criteria were published previously [25]. Table 1 shows subject demographics, cognitive test results, sample collection, and APOε genotype for the 24 aMCI subjects. Of these, 17 had CSF, peripheral blood (PB), and PET imaging; 3 had CSF and PB but no PET imaging; 3 had PB and PET imaging but no CSF; 2 had PET imaging and CSF but no PB; and 1 had PB but no CSF or PET (subject 702).

**Table 1:** Subject demographics

						Sample collection			
Subject number	Age/sex	CDR	MMSE	Immediate recall	Delayed recall	CSF	PB	PET (mean SUVR)	Genotype (ε3 or ε4)
105	59 M	0.5	29	11	9	Y	Y	NA	NA
702	70 F	0.5	29	8	8	NA	Y	NA	NA
715	71 F	0.5	29	7	6	Y	Y	NA	3–4
716	70 M	0.5	30	11	9	Y	Y	NA	3–3
718	65 F	0.5	28	14	13	Y	NA	1.25	3–4
721	75 M	0.5	30	16	10	NA	Y	1.20	2–3
727	66 F	0.5	28	10	9	Y	Y	1.18	NA
732	58 F	0.5	30	12	10	Y	Y	1.26	3–4
733	59 M	0.5	30	13	11	NA	Y	1.21	3–3
734	76 F	0.5	29	13	10	Y	Y	1.22	3–4
735	74 F	0.5	30	7	7	NA	Y	1.16	3–3
739	65 M	0.5	30	13	11	Y	Y	1.24	3–3
745	73 M	0.5	29	12	7	Y	NA	1.55	3–4
751	66 M	0.5	29	9	9	Y	Y	1.20	3–3
757	62 F	0.5	29	14	14	Y	Y	1.22	3–3
767	58 F	0.5	30	11	10	Y	Y	1.16	3–3
768	60 F	0.5	30	9	9	Y	Y	1.09	NA
771	70 M	0.5	30	11	11	Y	Y	1.09	3–3
782	55 F	0.5	28	11	9	Y	Y	1.08	3–3
787	62 M	0.5	30	12	11	Y	Y	1.11	3–4
794	61 M	0.5	30	10	10	Y	Y	1.21	NA
795	70 M	0.5	29	11	11	Y	Y	1.27	3–3
829	68 M	0.5	30	9	7	Y	Y	1.20	NA
831	64 F	0.5	29	8	8	Y	Y	1.19	3–4

Age is at time of sample collection

*CDR* Clinical Dementia Rating, *MMSE* Mini-Mental State Exam, *CSF* cerebrospinal fluid, *PB* peripheral blood, *PET* positron emission tomography, *SUVR* standardized uptake value ratio, genotype shown for APOε status: *Y* yes, *NA* not available

### CSF/peripheral blood lymphocytes collection and analysis

Sample collections occurred at the UT Southwestern Alzheimer’s Disease Center, consistent with the NIA Biospecimen Best Practices Guidelines, using established protocols [17]. Most sample collection was performed in the morning and initiation of sample processing occurred within 60 min of sample collection. Peripheral blood mononuclear cells (PBMC) were obtained via centrifugal Ficoll-based separation. CSF cells were obtained by centrifuging the CSF for 10-min at 394×*g* (4 °C) to collect the cell pellet at the bottom of the tube. The first 1 mL of CSF obtained during the lumbar puncture was discarded to minimize red blood cell contamination in the sample. CSF samples tinged red were not used. PBMCs and CSF cells were immediately stained with the fluorescent markers in parallel, and the resulting flow cytometry data was also acquired in parallel. The remaining PBMCs not used in the flow cytometry experiment were cryopreserved in media containing 50% human serum on the day of collection. This remaining PBMC sample was used for the APOε3/ε4 genotyping. CSF supernatant, blood plasma, and serum were aliquoted and stored at −80 °C for enzyme-linked immunosorbent assay (ELISA).

### Flow cytometry

CSF cells and PBMCs were resuspended with ice-cold FACS buffer (1× PBS, 4% BSA), and the cells were counted by a hemocytometer and verified by two team members, then stained with a multiplex panel consisting of CD45, CD4, CD8, CD19, CD27, and CD138 (BD Biosciences, San Jose, CA, USA) in parallel. CD3 was added to the flow panel after study of the first three aMCI subjects. No stains were used for live/dead cell exclusion since the cells were processed within an hour of collection. Gating strategies are shown in Additional file 1: Figure S1 and are based on PBMC events. Cells were incubated on ice in the dark, washed once, resuspended in 1 mL FACS buffer, preserved (4% paraformaldehyde), and obtained within 3 days on a FACS Aria (BD Biosciences). Flow cytometry data were analyzed with Flowjo (Tree Star). All immune cell subtype frequencies were normalized to the CD45^+^ leukocyte gate to allow for comparison of the immune profile across samples. The innate cell population is defined as CD45^+^CD3^−^CD19^−^ [17]. Data was acquired from all CSF cells obtained in the collection, and no minimum number of CSF cells was required to perform the flow cytometry experiments.

### ELISA

Quantitation of antibody isotypes (IgG and IgM) and cytokines (VEGF, IL17, BDNF, IL-10, Il-6, and IFNγ) by ELISA was performed as previously published [17]. Only IgG and IL-6 values are utilized in this study as the others did not display correlations with other parameters collected. For quantification of Aβ_42_, Innotest β-amyloid (1–42) ELISAs were performed as per manufacturer’s instructions (Fujirebio, Malvern, PA, USA).

### APOε genotyping

For genotype analysis, one million PBMCs were thawed and DNA was extracted using the DNeasy Blood and Tissue kit (Qiagen) as per the manufacturer’s instructions. APOε genotype was performed using TaqMan SNP genotyping assays C904973_10 (rs7412) and C_3084793_20 (rs429358; Life Technologies). The genotype was performed by the DNA Genotyping Core at UT Southwestern Medical Center using an ABI 7900HT PCR instrument according to the manufacturer’s instructions with modifications. Briefly, 10–20 ng/μL (3 μL) of DNA was added to each 96-well reaction plate containing 2.5 μL of reaction mix (2.5 μL TaqMan Master Mix, 0.225 μL ABI Probe (20×)). Allelic discriminations were performed using the thermal protocol of 50 °C for 2 min, 39 cycles of 95 °C for 15 s and 60 °C for 90 s, followed by a hold at 27 °C for 5 min. Genotype calls were determined using ABI Software SDS2.4.

### PET imaging

Participants were injected with a bolus of 10-mCi ^18^F-florbetapir 30 min prior to positioning in a Siemens ECAT HR PET scanner for a 10-min emission and 10-min transmission scan. A 2-min scout was acquired to ensure field of view without rotation in either the transverse or sagittal plane. At 50-min post-injection, two frames of 5-min PET emission scan and a 7-min transmission scan were acquired in a 3D mode using the following parameters: matrix size = 128 × 128, resolution at the image center = 5 × 5 mm, slice thickness = 2.42 mm, and field of view = 58.3 cm. Emission images were processed by iterative reconstruction, 4 iterations and 16 subsets with a 3-mm full width at half maximum (FWHM) ramp filter. The transmission image was reconstructed using back projection and a 6-mm FWHM Gaussian filter. Each PET image was spatially normalized to florbetapir uptake template (2 × 2 × 2 mm^3^ voxels) using SPM8 (Wellcome Trust Centre for Neuroimaging, London, UK) and in-house MATLAB (Mathworks Inc., Natick, MA) scripts and visually inspected for registration quality. Standardized uptake value ratio (SUVR) was computed using mean cerebellar uptake as reference. Three regions-of-interest (ROI) relevant to AD pathology were selected a priori: posterior cingulate (PCC), precuneus, and mean cortex. The mean cortical SUVR was calculated by taking an average of posterior and anterior cingulate, precuneus, temporal, dorsolateral prefrontal, orbital frontal, parietal, and occipital SUVRs [25].

### Statistical analysis

All data are reported as mean ± standard deviation, with statistical significance set a priori at *p* < 0.05 for all tests. A non-parametric Mann-Whitney test was used to compare immune cell population frequencies between APOε3 and APOε4 carriers. Spearman rank order correlations were performed to assess simple linear correlation for parameters including PET imaging, immune cell populations, and ELISA data. Multiple comparison correction was not performed for this exploratory study. All statistical analyses were performed using SPSS 20 (SPSS Inc.; Chicago, IL) and GraphPad Prism (La Jolla, CA).

## Results

### Cortical Aβ deposition coincides with decreased soluble Aβ_42_ in the CSF

**Fig. 1:**
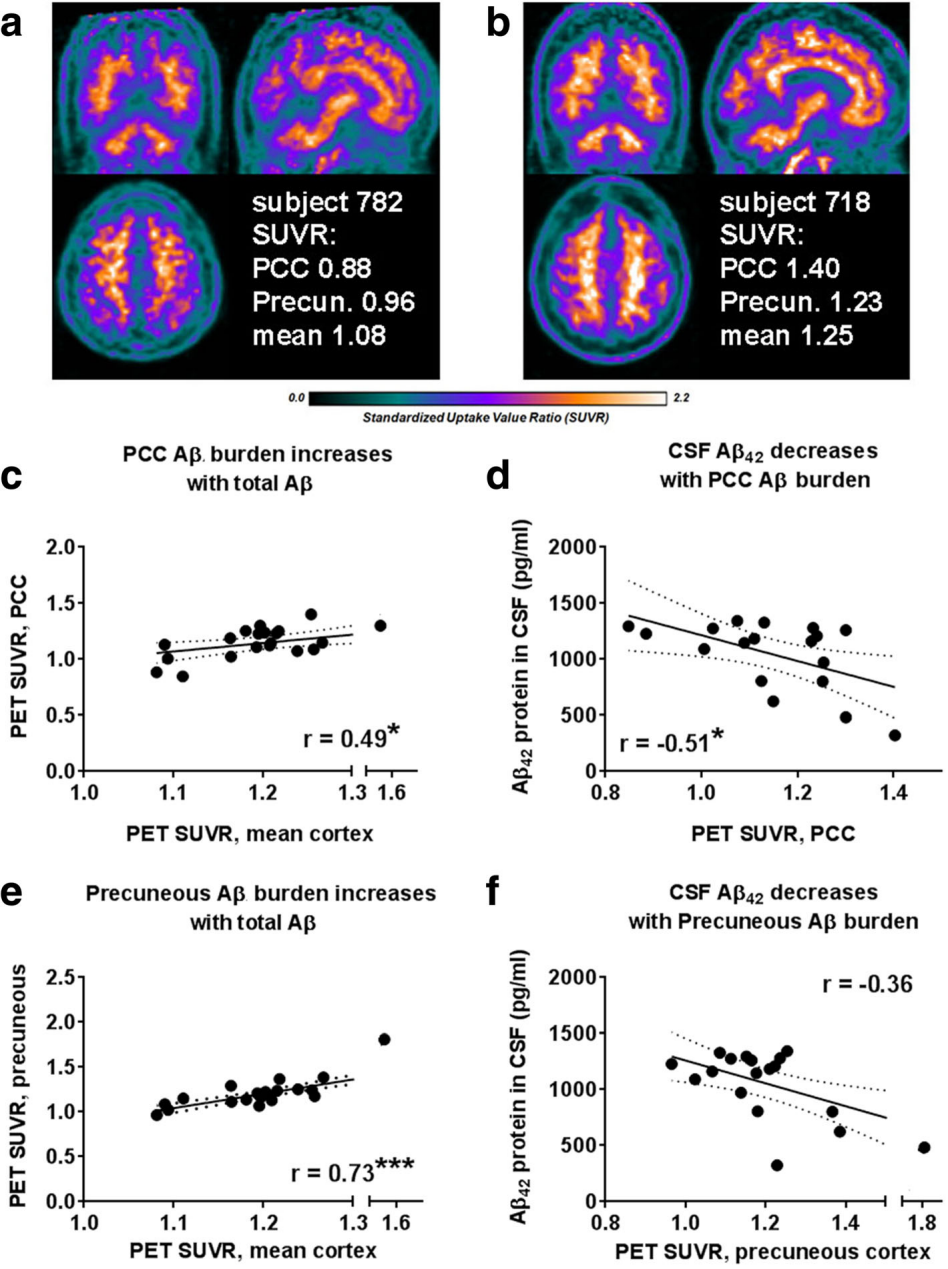
PET reflects dysregulation of Aβ clearance. **a**, **b** Representative PET images and values corresponding to the standardized uptake value ratio (SUVR), with image of the posterior cingulate cortex (PCC). **c** Increases in PCC SUVR predict total amyloid burden (mean cortex). **d** PCC SUVR increase corresponds to a decrease in soluble CSF Aβ_42_. **e**, **f** Increases in precuneous cortex SUVR also predict total amyloid burden, but without the significant decrease in soluble CSF Aβ_42_. Linear regression (*solid lines*) and 95% confidence interval (*dotted lines*) shown. *p < 0.05, ***p < 0.001

Figure 1 depicts representative PET imaging of cortical Aβ deposition in two aMCI subjects, including values for PCC and precuneous cortex, as early regions for amyloid deposition in AD [26, 27]. Mean SUVR amyloid burden for the entire brain (represented as “PET SUVR mean”) of each subject is listed in Table 1. All Spearman’s rho values and *p* values for correlations are shown in Table 2, and larger group PET data previously published [25].

**Table 2:** Spearman rank order correlations for PET amyloid deposition and immune profile in aMCI subjects

					Cerebrospinal fluid (CSF)										Peripheral blood (PB)		
	Age	PET-PCC	PET-precuneous	PET-mean	Aβ_42_	IL6	IgG	CD4 T cell %	B cell %	Memory B cell%	Plasmab %	T cell #	CD4 T cell #	CD4:CD8	CD4 T cell%	B cell %	CD4:CD8
Age		0.66**	0.54*	0.20	−0.20	−0.63*	−0.22	**−** 0.49*	0.29	0.39	0.60**	−0.28	−0.30	−0.39	0.10	−0.32	0.07
PET-PCC	*(0.002)*		0.49*	0.49*	−0.51*	−0.66*	0.20	−0.35	0.25	0.16	0.28	−0.57*	**−** 0.54*	−0.20	0.16	−0.28	0.12
PET-precuneous	*(0.013)*	*(0.029)*		0.73**	−0.36	−0.45	0.002	−0.39	0.10	0.38	0.39	−0.12	−0.21	−0.22	−0.34	0.58**	−0.32
PET-mean	(0.39)	*(0.027)*	*(<0.0001)*		−0.52*	−0.06	0.04	−0.31	0.09	0.56*	0.18	−0.13	−0.22	−0.05	−0.33	−0.43	−0.35
CSF | Aβ_42_	(0.38)	*(0.030)*	(0.14)	*(0.029)*		0.04	−0.45*	0.17	−0.17	−0.40	−0.23	0.35	0.39	0.36	0.17	0.23	0.25
CSF | IL6	*(0.027)*	*(0.038)*	(0.19)	(0.88)	(0.90)		0.17	0.68	−0.02	−0.51	−0.52	0.16	0.13	0.34	0.01	0.57	0.14
CSF | IgG	(0.33)	(0.44)	(0.99)	(0.88)	*(0.04)*	(0.62)		−0.10	−0.19	−0.39	−0.16	−0.35	−0.25	−0.01	0.29	−0.06	0.22
CSF | CD4 T cell (%)	*(0.047)*	(0.22)	(0.17)	(0.28)	(0.52)	(0.86)	(0.70)		−0.46	−0.05	−0.21	0.47	0.59*	0.67**	−0.09	0.28	−0.08
CSF | B cell (%)	(0.21)	(0.34)	(0.69)	(0.72)	(0.46)	(0.95)	(0.44)	(0.06)		−0.02	0.17	−0.47	−0.56*	−0.18	0.09	−0.16	−0.01
CSF | memory B (%)	(0.09)	(0.54)	(0.13)	*(0.02)*	(0.08)	(0.11)	(0.10)	(0.85)	(0.93)		0.37	0.08	0.01	−0.32	−0.54*	−0.34	**−** 0.63**
CSF | plasmablasts (%)	*(0.005)*	(0.28)	(0.12)	(0.49)	(0.33)	(0.10)	(0.50)	(0.42)	(0.47)	(0.11)		−0.04	−0.08	−0.49*	0.09	−0.43	−0.08
CSF | T cell (#)	(0.27)	*(0.034)*	(0.68)	(0.65)	(0.17)	(0.68)	(0.17)	(0.06)	(0.06)	(0.76)	(0.87)		0.95**	0.37	−0.21	0.30	−0.08
CSF | CD4 T cell (#)	(0.25)	*(0.046)*	(0.47)	(0.44)	(0.12)	(0.74)	(0.34)	*(0.01)*	*(0.019)*	(0.99)	(0.75)	*(<0.0001)*		0.50*	−0.14	0.09	−0.03
CSF | CD4:CD8	(0.13)	(0.49)	(0.45)	(0.86)	(0.15)	(0.37)	(0.97)	*(0.003)*	(0.49)	(0.20)	(0.05)	(0.14)	*(0.04)*		0.13	0.17	0.19
PB | CD4T (%)	(0.68)	(0.56)	(0.20)	(0.22)	(0.48)	(0.97)	(0.22)	(0.73)	(0.72)	*(0.026)*	(0.74)	(0.41)	(0.60)	(0.63)		0.20	0.94**
PB | B cell (%)	(0.13)	(0.23)	*(0.007)*	(0.06)	(0.30)	(0.05)	(0.79)	(0.28)	(0.49)	(0.14)	(0.06)	(0.24)	(0.72)	(0.51)	(0.40)		0.18
PB | CD4:CD8	(0.76)	(0.65)	(0.22)	(0.19)	(0.31)	(0.70)	(0.37)	(0.75)	(0.99)	*(0.007)*	(0.77)	(0.76)	(0.91)	(0.45)	*(<0.0001)*		0.18

In the rho-value section of the table, rho-values that are significant are in italics and * = <0.05 and ** = <0.01. Specific p-values are in parenthesis with significant values in italics

Amyloid deposition in the PCC and precuneous cortex occur early in AD patients [4, 6] and can predict conversion of aMCI to AD [28]. In the aMCI cohort presented here, total cortical Aβ burden as measured by PET correlated with PCC SUVR (*r* = 0.49; *p* < 0.05; Fig. 1c). However, total cortical Aβ burden as measured by PET correlated even more strongly with precuneous SUVR (*r* = 0.73; *p* < 0.001, Fig. 1e). CSF Aβ_42_ levels were associated inversely with PCC Aβ burden (*r* = −0.51, *p* < 0.05; Fig. 1d), but an inverted correlation of CSF Aβ_42_ levels with precuneous SUVR did not reach significance (*r* = −0.36, *p* > 0.05). These data confirm that even in aMCI patients, Aβ deposition in the PCC (but not in the precuneous region) associates with loss of soluble Aβ_42_ in the CSF.

### Reductions in CD4 T cells in the CSF positively associated with Aβ deposition in the PCC

**Fig. 2:**
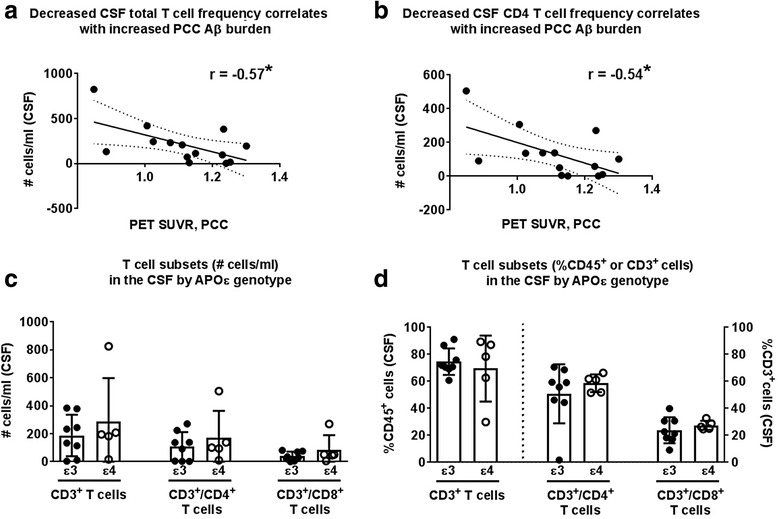
T cell numbers in the CSF decrease with increased amyloid burden. Both the **a** total number of T cells in the cerebrospinal fluid (CSF) and **b** the number of CD4+ T cells in the CSF decreases as amyloid burden identified by PET standardized uptake value ratio (SUVR) in the posterior cingulate cortex (PCC) increases. **c**, **d** APOε status does not affect T cell populations numbers or percent representation in the CSF. Linear regression (*solid lines*) and 95% confidence interval (*dotted lines*) shown. *p < 0.05

Others have reported that T cells are one critical component of Aβ_42_ clearance [29]. Therefore, we examined the relationship between T cell frequency and Aβ deposition. We found that total T cells, and specifically the CD4 T cell subtype, were inversely associated with increased PCC Aβ burden (T cells: *r* = −0.57, *p* < 0.05; CD4+ T cells: *r* = −0.54, *p* < 0.05; Fig. 2a, b). There was no similar correlation within the CD8 T cell populations in either the CSF or periphery, or for the CD4:CD8 T cell ratio (Table 2). Innate cell populations also did not correlate to age or Aβ burden in either the CSF or peripheral blood compartments (data not presented, but found in Additional file 2). As exacerbated disruption of Aβ_42_ clearance mechanisms have been observed in APOε4 carriers [8] who are also at a greater risk for AD [30–32], we also examined T cell frequencies and Aβ deposition in APOε4 carriers and non-carriers (APOε3+). When considering genotype, we did not observe a generalized effect of APOε status on T cell populations in the CSF (Fig. 2c, d) or peripheral blood. Taken together, these data suggest a reduction in circulating CD4 T cells within the CSF is coincident with an accumulation of PCC Aβ independent of ApoE genotype.

### B cells shift from the periphery to the CSF as Aβ burden increases

**Fig. 3:**
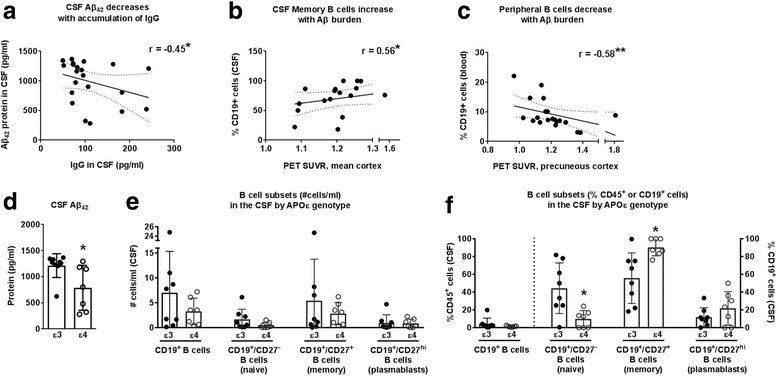
B cells shift from the periphery to the CSF as amyloid burden increases. **a** Soluble IgG in the cerebrospinal fluid (CSF) declines with loss of soluble CSF Aβ_42_. **b** Increased mean standardized uptake value ratio (SUVR) for the total cortex correlates to an increase in memory B cell percent representation in the CSF, while **c** increased Aβ deposition in the precuneous cortex associates with a decline in B cell representation in the peripheral blood. **d** Soluble Aβ_42_ in the cerebrospinal fluid (CSF) is decreased in APOε4+ subjects. **e**, **f** Apoε4 carriers exhibit enhanced memory B cell percent representation in the CSF, but genotype did not affect overall B cell distribution

As the other main effector cell of the adaptive immune system, we also investigated B cells subsets (i.e., naive, memory, and plasmablasts) in the CSF and peripheral blood, as well as immunoglobulin (Ig)G levels. Surprisingly, CSF levels of IgG (*r* = −0.45, *p* < 0.05; Fig. 3a) were inversely associated with soluble Aβ_42_ in the CSF, suggesting that antibody accumulation in the CSF is coincident with the dysregulation of amyloid clearance. Within the CSF, the frequency of memory B cells positively associated with increased Aβ burden (*r* = 0.56; *p* < 0.05; Fig. 3b). In contrast, peripheral blood B cells were inversely associated with precuneous Aβ burden (*r* = −0.58, *p* < 0.01; Fig. 3c), though specific peripheral naïve and plasmablast B cell subsets remained unaffected by Aβ burden. Interestingly, the decline in peripheral memory B cells is associated with a decline in delayed recall (*r* = −0.46; *p* < 0.05). Finally, CSF interleukin (IL)-6 was inversely associated with PCC Aβ burden (*r* = −0.66, *p* < 0.05; Table 2).

APOε4 may also affect B cell function, as B cells highly express the APOε receptors necessary for APOε clearance of Aβ [33, 34]. We found that APOε4 carriers had significantly lower CSF Aβ_42_ compared to APOε3 subjects (APOε3^+^: 1214 ± 227 pg/mL vs. APOε4^+^: 788 ± 430 pg/mL; *p* < 0.05; Fig. 3d). We compared B cell subtype frequencies in APOε4 and APOε3 carriers and found no statistical difference between B cell numbers (Fig. 3e). However, APOε4 carriers exhibited a significantly lower frequency of naïve B cells, and a higher frequency of memory B cells, in the CSF (Fig. 3f). This effect of genotype was not observed in the peripheral B cell pools. Thus, the loss of B cells from the periphery, and the gain of memory B cells in the CSF, is highly suggestive of an adaptive humoral immune response concomitant with increased Aβ burden.

## Discussion

While Aβ burden is a hallmark of AD, we are only beginning to understand the impact of neuroinflammation on AD pathology. To this end, we and others have investigated the immune profile of patients at high risk to develop AD and reported potential involvement of multiple inflammatory components in AD onset and progression [17, 29]. However, given that Aβ deposition is a common feature of AD, it stands to reason that there may be a relationship between Aβ deposition and the immune profile as part of an endogenous immune response associated with early AD pathology. Since the interplay of these two factors remains largely unknown, we investigated the relationship between PET Aβ imaging, CSF Aβ_42_ levels, and the immune profiles in the CSF and blood of a well-documented cohort of aMCI patients [17, 25] prodromal for AD. Dysregulation of Aβ_42_ clearance is thought to occur ~10 years preceding AD diagnosis [8] and likely to be exacerbated in aMCI. Aβ_42_ production is also reported to be proportional to neuronal activity and highest in the PCC and precuneus cortex, which are components of the default-mode network (DMN) and affected in AD [26, 27, 35, 36]. In addition, higher Aβ burden in the precuneous cortex most accurately predicts conversion of aMCI to AD [28]. Therefore, we focused on these regions for our analyses.

**Fig. 4:**
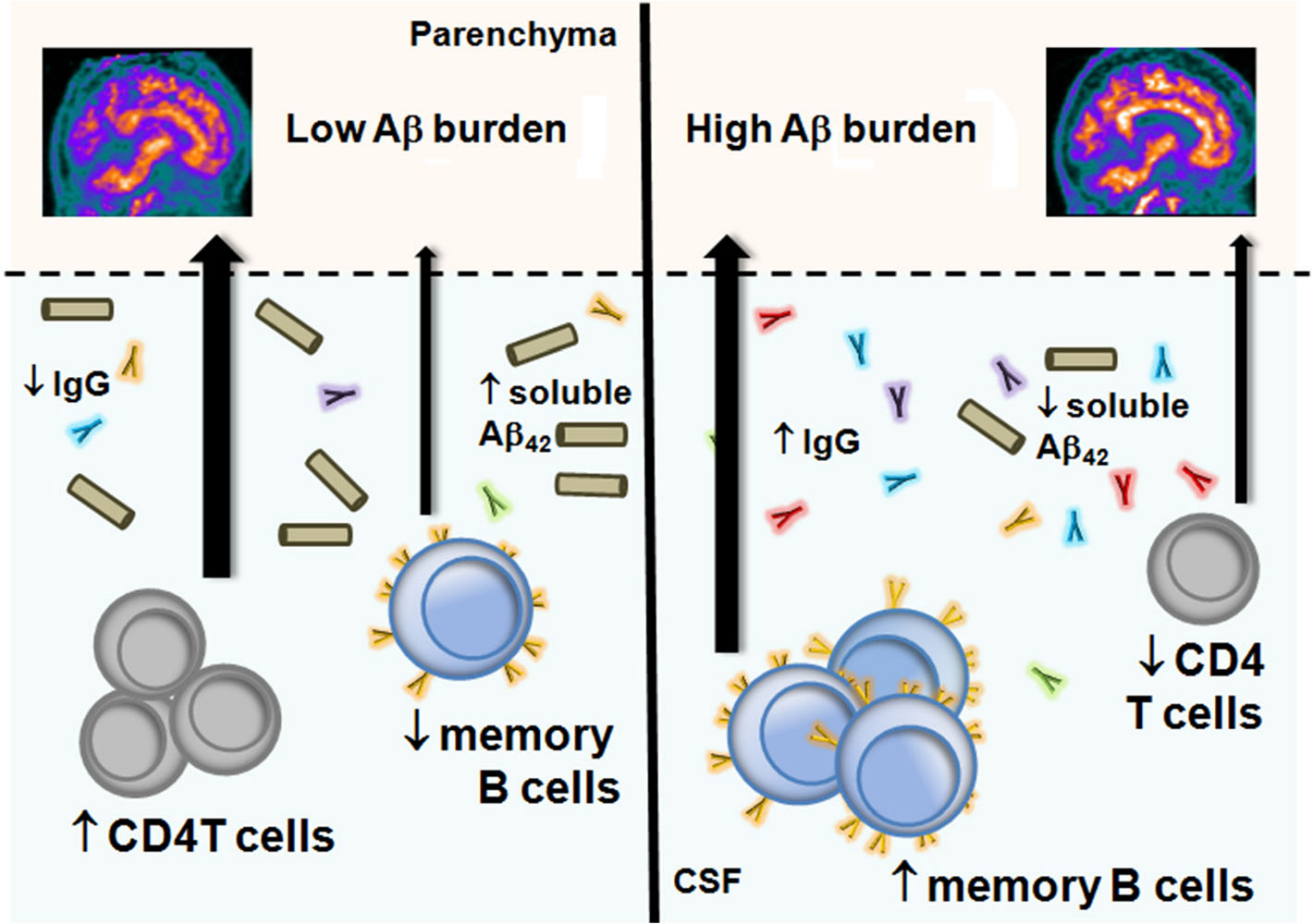
Summary of adaptive immune changes in the CSF with amyloid deposition. This figure highlights the findings that CSF lymphocyte populations shift from high CD4 T cells to high memory B cells with increased Aβ deposition in the brain. This also includes a reversal the IgG:soluble Aβ_42_ levels with increased amyloid burden

Within the T cell populations in the CSF of aMCI subjects, reduction in the CD4 T cell frequency is associated with increased Aβ burden (summarized in Fig. 4). This observation may reflect the loss of T cell activation and expansion and corroborates our previous findings in a smaller cohort of aMCI patients in which we observed a reduction of CD4+ T cells in the CSF in comparison to younger patients with a known CD4 T cell-mediated neurological disease of the CNS [17]. Despite this correlation of reduced T cell frequency and increased Aβ burden, we did not find any significant relation between T cell frequency and global cognitive or memory function in our aMCI cohort as assessed by MMSE and immediate and delayed recall (Additional file 2). Others have also observed no correlation of activated CD4 T cells with a battery of cognitive domain tests in aMCI subjects (*n* = 19) [29]. It is thought that T cells in the CSF would typically mediate an Aβ_42_-directed immune response to clear amyloid from the brain [37], which should result in improved cognition and brain health. However, the aMCI cohorts in our study or the Lueg study do not support this concept. It is possible that such correlations cannot be observed except in more advanced disease, and indeed, in the Lueg study, activated CD8 T cells correlated with neurocognitive deficits in AD subjects. These results highlight the need for longitudinal multi-center investigations of the immune profiles of aMCI subjects in order to understand the potential roles of T cells in the advancement of this devastating disease.

Increased cortical Aβ burden in our aMCI cohort correlated with decreased frequency of peripheral CD19+ B cells, similar to other results in AD [22]. This finding suggests that egress of B cells from the peripheral compartment may be an early event associated with AD development and emphasizes an urgent need to identify critical factors that may mediate egress of B cells into the CNS. Even more importantly, cortical Aβ burden correlated with a shift from naïve to memory B cell subsets in the CSF, independent of age (summarized in Fig. 4). Naive B cells proliferate and differentiate into memory B cells upon initial exposure to their cognate antigen. These memory B cells circulate indefinitely until subsequent encounter with their cognate antigen elicits a rapid and enhanced adaptive immune response [38]. Although the antigen(s) driving this shift to memory B cells in the CSF of aMCI patients remains unknown, it is possible that the memory B cell pool is enriched for Aβ_42_-reactive B cells that are long-lived and poised for rapid differentiation to antibody secreting plasmablasts and plasma cells.

Re-activation of memory B cells following antigen exposure induces conversion to plasmablasts and plasma cells that secrete antibody, which may be important for Aβ clearance [39, 40]. In fact, the intent of potential therapies of active Aβ immunization is to promote the production of anti-Aβ antibodies by resident plasmablasts and plasma cells [41–43]. The role of plasmablasts in Aβ clearance remains unclear, although we did observe a significant correlation between the frequency of plasmablasts in the CSF and amyloid depostion (Supplemental data). Interestingly, the plasmablast survival factor, IL-6 [44], was also decreased in the CSF supernatants of our aMCI cohort and this decrease correlates with increased Aβ burden. This finding could indicate either impaired ability for various cell types of aMCI patients to produce IL-6, or that emerging plasmablasts are rapidly consuming available IL-6. Of note, memory B cells display an increased IL-6 output with advancing age [44], but we could not verify this finding by others as the small sample size for our exploratory cohort precludes controlling our results by subject age. The consequences of IL-6 availability and impact of this antibody secreting B cell subtype in AD neuropathology or neuroprotection requires further investigation in a cohort of patients powered to control for possible age-related confounders.

It is well-established that the APOε4 genotype is associated with Aβ burden in AD [45]. It has also been known since 1998 that B cells express the low-density lipoprotein (LDL) receptor (LDLR) [34], the main receptor for cholesterol metabolism that binds APOε3 and APOε4 to modulate inflammation [46–48]. Peripheral B cells are capable of internalizing and degrading LDL, with an increased capacity for degradation upon immune activation [34]. But while B cells express the APOε receptors necessary for APOε-mediated clearance of Aβ from the brain [33, 34], there are no studies investigating the effect of APOε genotype on endogenous B cell function. We found that the frequency of antigen-experienced memory B cells in the CSF was elevated in APOε4^+^ aMCI patients compared to APOε3^+^ aMCI patients. Yet APOε4^+^ aMCI patients also displayed decreased CSF Aβ_42_ in comparison to APOε3^+^ aMCI patients. These observations may indicate that the humoral immune response against Aβ_42_ is defective in the at-risk CNS or that the memory B cell pool is responding to other factors enriched in the at-risk CNS that would cause their expansion in a bystander-like fashion.

Finally, innate cells, operating as antigen-presenting cells (APC), are required to activate adaptive immune components, including T cells and B cells [47, 49]. This emphasizes the profound impact defective innate cells can have on adaptive immunity, including amyloid clearance [50]. We found that CD45^+^ leukocytes and pro-inflammatory cytokines were present in the CSF of aMCI patients, at levels equivalent to or exceeding those found in early MS patients [17], a prototypical inflammatory CNS disease [51]. Innate cell population frequencies did not, however, correlate with amyloid deposition. Unfortunately, our flow cytometry panel precludes identification of specific innate cell subsets. Others have not found differences in monocytes, NK cells, and NK T cells in aMCI or AD patients compared to healthy controls [29], but the specific relationship remains to be determined with regard to the AD pathology as revealed by PET amyloid imaging.

## Conclusions

A recent AD review [52] called for targeted investigation of potentially beneficial CNS-directed autoreactivity that may mediate protection from neurodegeneration. However, the role of amyloid deposition in the progression of AD, or in relation to neuroinflammation, remains in question, with arguments both supporting [53] and rejecting [54] a foundational role for Aβ_42_ deposition in the initiation and/or progression of AD. Despite the limitations of a small sample size and cross-sectional nature of the present study, our data reveal several potentially important relationships between the adaptive immune system and brain amyloid deposition in patients with aMCI. Future studies are warranted to reliably quantify changes in leukocyte populations with regard to both brain Aβ burden using PET amyloid imaging, as well as stratifications by APOε genotype. Mechanistic studies are also needed to determine whether dysregulation of the immune system contributes to the increase of amyloid burden in and/or Aβ_42_ clearance from the CNS.

## Additional files

Additional file 1: Figure S1. Gating strategy for lymphocyte analysis in the blood and CSF. Top row shows initial gating strategies to identify live cells from FACS, which are further subdivided as CD45+ leukocytes (row 2). CD45+ populations are parsed into CD3+ T cell populations (right side) and CD19+ B cell populations (left side), with individual lymphocyte subsets identified. (JPG 295 kb)

Additional file 2: Complete table of all Spearman rank order correlations performed for the manuscript. (XLSX 88 kb)

## Abbreviations

AD: Alzheimer’s disease
aMCI: Amnestic mild cognitive impairment
APOε: Apolipoprotein E
Aβ: Amyloid beta
CDR: Clinical Dementia Rating
CNS: Central nervous system
CSF: Cerebrospinal fluid
DMN: Default-mode network
ELISA: Enzyme-linked immunosorbent assay
IL: Interleukin
MMSE: Mini-Mental State Exam
MS: Multiple sclerosis
PB: Peripheral blood
PBMC: Peripheral blood mononuclear cells
PCC: Posterior cingulate
PET: Positron emission tomography
SUVR: Standardized uptake value ratio
UTSW: UT Southwestern

## References

[1] Middleton LE (2009). Promising strategies for the prevention of dementia. Arch Neurol.

[2] Richard E (2012). Methodological challenges in designing dementia prevention trials—the European Dementia Prevention Initiative (EDPI). J Neurol Sci.

[3] Kling MA (2013). Vascular disease and dementias: paradigm shifts to drive research in new directions. Alzheimers Dement.

[4] Morris JK, et al. Is Alzheimer’s disease a systemic disease?, in Biochim Biophys Acta. 2014;1842(9):1340–1349. 10.1016/j.bbadis.2014.04.012 PMC4126236 24747741

[5] Rodrigue KM (2012). beta-Amyloid burden in healthy aging: regional distribution and cognitive consequences. Neurology.

[6] Villemagne VL (2013). Amyloid beta deposition, neurodegeneration, and cognitive decline in sporadic Alzheimer’s disease: a prospective cohort study. Lancet Neurol.

[7] Storandt M (2009). Cognitive decline and brain volume loss as signatures of cerebral amyloid-beta peptide deposition identified with Pittsburgh compound B: cognitive decline associated with Abeta deposition. Arch Neurol.

[8] Mawuenyega KG (2010). Decreased clearance of CNS beta-amyloid in Alzheimer’s disease. Science.

[9] Iwata N (2000). Identification of the major Abeta1-42-degrading catabolic pathway in brain parenchyma: suppression leads to biochemical and pathological deposition. Nat Med.

[10] Shibata M (2000). Clearance of Alzheimer’s amyloid-ss (1–40) peptide from brain by LDL receptor-related protein-1 at the blood-brain barrier. J Clin Invest.

[11] Iliff JJ, et al. A paravascular pathway facilitates CSF flow through the brain parenchyma and the clearance of interstitial solutes, including amyloid beta. Sci Transl Med. 2012;4(147):147ra111. 10.1126/scitranslmed.3003748 PMC3551275 22896675

[12] Akiyama H (2000). Inflammation and Alzheimer’s disease. Neurobiol Aging.

[13] Wyss-Coray T (2002). Inflammation in neurodegenerative disease—a double-edged sword. Neuron.

[14] Querfurth HW (2010). Alzheimer’s disease. N Engl J Med.

[15] Tierney MC (1996). Prediction of probable Alzheimer’s disease in memory-impaired patients: a prospective longitudinal study. Neurology.

[16] Bowen J (1997). Progression to dementia in patients with isolated memory loss. Lancet.

[17] Monson NL (2014). Elevated CNS inflammation in patients with preclinical Alzheimer’s disease. J Cereb Blood Flow Metab.

[18] Larbi A (2009). Dramatic shifts in circulating CD4 but not CD8 T cell subsets in mild Alzheimer’s disease. J Alzheimers Dis.

[19] Pellicano M (2012). Immune profiling of Alzheimer patients. J Neuroimmunol.

[20] Speciale L (2007). Lymphocyte subset patterns and cytokine production in Alzheimer’s disease patients. Neurobiol Aging.

[21] Lombardi VR (1999). Characterization of cytokine production, screening of lymphocyte subset patterns and in vitro apoptosis in healthy and Alzheimer’s disease (AD) individuals. J Neuroimmunol.

[22] Richartz-Salzburger E (2007). Altered lymphocyte distribution in Alzheimer’s disease. J Psychiatr Res.

[23] Clark CM (2011). Use of florbetapir-PET for imaging beta-amyloid pathology. JAMA.

[24] Selkoe DJ (2012). Preventing Alzheimer’s disease. Science.

[25] Tarumi T (2015). Amyloid burden and sleep blood pressure in amnestic mild cognitive impairment. Neurology.

[26] Bero AW (2011). Neuronal activity regulates the regional vulnerability to amyloid-beta deposition. Nat Neurosci.

[27] Buckner RL (2005). Molecular, structural, and functional characterization of Alzheimer’s disease: evidence for a relationship between default activity, amyloid, and memory. J Neurosci.

[28] Hatashita S (2013). Diagnosed mild cognitive impairment due to Alzheimer’s disease with PET biomarkers of beta amyloid and neuronal dysfunction. Plos One.

[29] Lueg G (2015). Clinical relevance of specific T-cell activation in the blood and cerebrospinal fluid of patients with mild Alzheimer’s disease. Neurobiol Aging.

[30] Adluru N (2014). White matter microstructure in late middle-age: effects of apolipoprotein E4 and parental family history of Alzheimer’s disease. Neuroimage Clin.

[31] Holtzman DM (2012). Apolipoprotein E and apolipoprotein E receptors: normal biology and roles in Alzheimer disease. Cold Spring Harb Perspect Med.

[32] Taylor JL (2014). APOE-epsilon4 and aging of medial temporal lobe gray matter in healthy adults older than 50 years. Neurobiol Aging.

[33] Fryer JD (2005). The low density lipoprotein receptor regulates the level of central nervous system human and murine apolipoprotein E but does not modify amyloid plaque pathology in PDAPP mice. J Biol Chem.

[34] De Sanctis JB (1998). Expression of low-density lipoprotein receptors in peripheral blood and tonsil B lymphocytes. Clin Exp Immunol.

[35] Cirrito JR (2005). Synaptic activity regulates interstitial fluid amyloid-beta levels in vivo. Neuron.

[36] Pfefferbaum A (2011). Cerebral blood flow in posterior cortical nodes of the default mode network decreases with task engagement but remains higher than in most brain regions. Cereb Cortex.

[37] Jozwik A (2012). Beta-amyloid peptides enhance the proliferative response of activated CD4CD28 lymphocytes from Alzheimer disease patients and from healthy elderly. Plos One.

[38] Shlomchik MJ (2012). Germinal center selection and the development of memory B and plasma cells. Immunol Rev.

[39] Ligocki AJ (2013). Expansion of CD27high plasmablasts in transverse myelitis patients that utilize VH4 and JH6 genes and undergo extensive somatic hypermutation. Genes Immun.

[40] Metcalf TU (2011). Alphavirus-induced encephalomyelitis: antibody-secreting cells and viral clearance from the nervous system. J Virol.

[41] Tarawneh R (2009). Critical issues for successful immunotherapy in Alzheimer’s disease: development of biomarkers and methods for early detection and intervention. CNS Neurol Disord Drug Targets.

[42] Zotova E (2013). Inflammatory components in human Alzheimer’s disease and after active amyloid-beta42 immunization. Brain.

[43] Wisniewski T (2014). Immunotherapy for Alzheimer’s disease. Biochem Pharmacol.

[44] Bancos S (2010). Memory B cells from older people express normal levels of cyclooxygenase-2 and produce higher levels of IL-6 and IL-10 upon in vitro activation. Cell Immunol.

[45] Drzezga A (2009). Effect of APOE genotype on amyloid plaque load and gray matter volume in Alzheimer disease. Neurology.

[46] Zhang H (2011). Cross-talk between apolipoprotein E and cytokines. Mediators Inflamm.

[47] Gale SC, et al. APOepsilon4 is associated with enhanced in vivo innate immune responses in human subjects. J Allergy Clin Immunol. 2014;134(1):127–34. 10.1016/j.jaci.2014.01.032 PMC4125509 24655576

[48] Grocott HP (2001). Apolipoprotein E genotype differentially influences the proinflammatory and anti-inflammatory response to cardiopulmonary bypass. J Thorac Cardiovasc Surg.

[49] Le Page A (2015). NK cells are activated in amnestic mild cognitive impairment but not in mild Alzheimer’s disease patients. J Alzheimers Dis.

[50] Fiala M (2007). Phagocytosis of amyloid-beta and inflammation: two faces of innate immunity in Alzheimer’s disease. J Alzheimers Dis.

[51] Ligocki AJ (2015). A distinct class of antibodies may be an indicator of gray matter autoimmunity in early and established relapsing remitting multiple sclerosis patients. ASN Neuro.

[52] Schwartz M (2014). Breaking peripheral immune tolerance to CNS antigens in neurodegenerative diseases: boosting autoimmunity to fight-off chronic neuroinflammation. J Autoimmun.

[53] Musiek ES (2015). Three dimensions of the amyloid hypothesis: time, space and ‘wingmen’. Nat Neurosci.

[54] Herrup K (2015). The case for rejecting the amyloid cascade hypothesis. Nat Neurosci.

