# Supplementary figures and images for: Adaptive lymphocyte profiles correlate to brain Aβ burden in patients with mild cognitive impairment
Source: J Neuroinflammation. 2017 Jul 27;14:149. doi: 10.1186/s12974-017-0910-x (PMC5530920; doi:10.1186/s12974-017-0910-x)

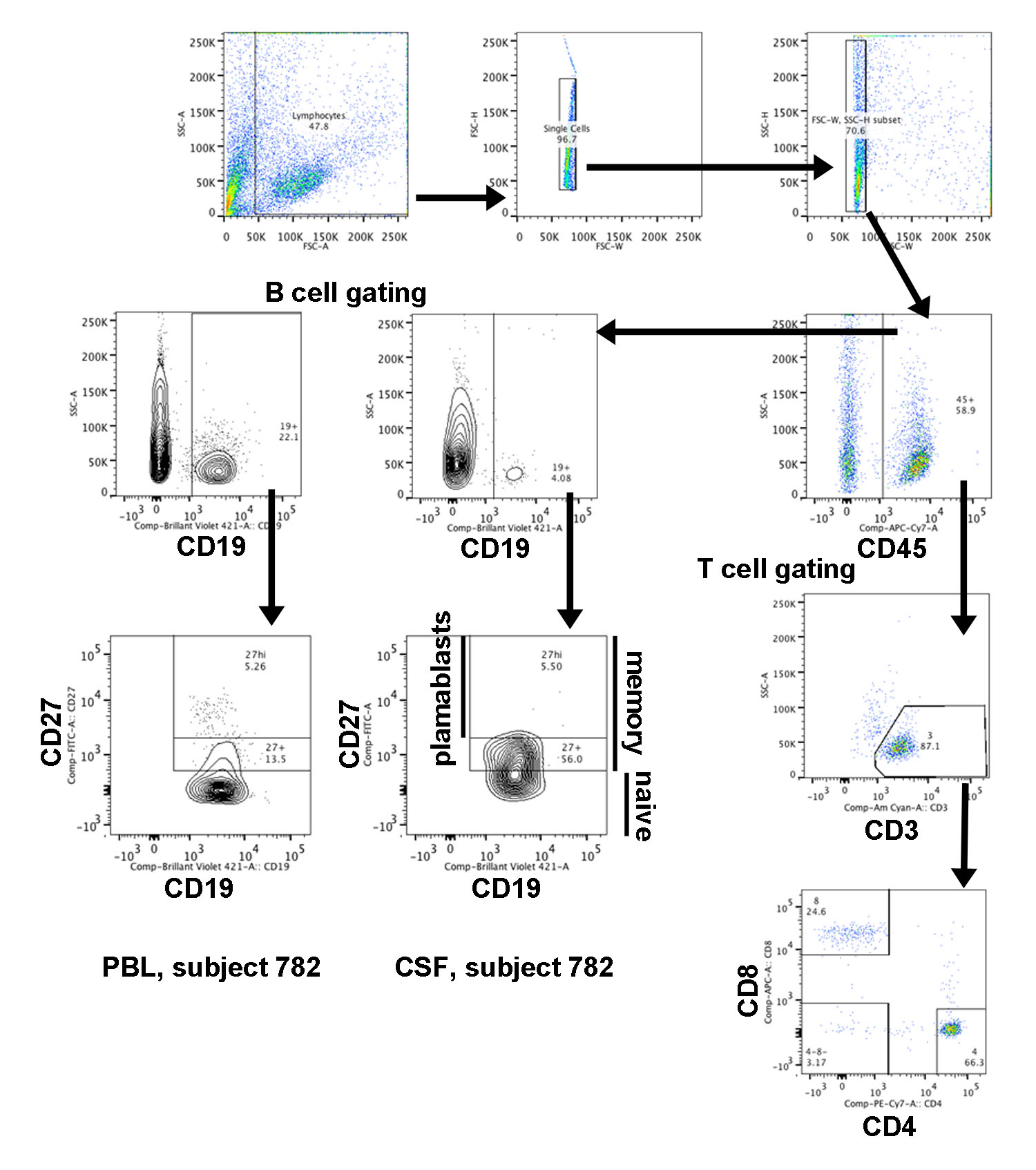

Supplement: Supplementary file 1 — Gating strategy for lymphocyte analysis in the blood and CSF. Top row shows initial gating strategies to identify live cells from FACS, which are further subdivided as CD45+ leukocytes (row 2). CD45+ populations are parsed into CD3+ T cell populations (right side) and CD19+ B cell populations (left side), with individual lymphocyte subsets identified. (JPG 295 kb) [file 12974_2017_910_MOESM1_ESM.jpg]
